# On Cysts of the Mouth

**Published:** 1879-08

**Authors:** 


### ARTICLE VI.

### *On Cysts of the Mouth.*

Mr. Francis Fox read a paper before the British Association of Dental Surgeons, on "Cysts of the Mouth." After alluding to the oral mucous membrane being not infrequently the seat of cystic disease, owing to the numerous small glands and follicles in this situation, and that the mere occlusion of the duct of one of these may give rise to the formation of a more or less extensive tumor, Mr. Fox observed that these cysts may be congenital, or may rise subsequent to birth, in consequence of some morbid action set up in the mucous membrane. The term "ranula" has been, and still is, applied to all cystic formations in the sublingual region, and he could not suggest a more appropriate appellation, provided we do not associate the name with any special pathological change, but regard it as simply descriptive of the impression which such tumors make upon the observer. Ranula, then, may depend upon (*a*) obstruction of the outflow from the minute follicles or conglobate glands; (*b*) it may be connected with the Whartonian, or one of the larger ducts, or (*c*) it may be in no way connected with the mucous structure itself; (*d*) it may depend upon inflammation of the bursa, which is placed between the genio-hyoglossi muscles. Similar cysts to these ranulæ occur in the mucous membrane of the lips and cheeks, and are often met with on mucous surfaces in other regions of the body. Their treatment is simple. If due to local inflammation, a small puncture will quickly effect a cure; or, if the orifice of the duct can be discovered, a small probe may be inserted; or if arising from a salivary calculus, an incision into the duct will permit the easy removal of the concretion. If the enlargement be excessive, a seton of a few threads of silk may be passed through the tumor, so that the contents will gradually escape, and inflammation be set up. This may give rise to an excessive amount of inflammation, and occasion much secondary mischief, and a better course of procedure might consequently be to puncture the tumor, and then apply caustic to its internal surface. It may be necessary to remove a portion of the cyst wall, in which case the more vascular portion of the tumor should be avoided. Ranula connected with the duct of the sub-maxillary gland is dependent upon dilatation of its coats, in consequence of obstruction at its orifice or in some part of its canal. Calcareous deposit is the commonest cause of obstruction, and these salivary calculi attain often a very great size, and are amenable to the same treatment already mentioned. There is still some difference of opinion with regard to the exact pathology of these sublingual cysts. Some maintain that they are invariably connected with the large salivary glands; others, that they occur quite independently of these ducts, and are, or nearly resemble, "mucous cysts." The fluid which they contain does not resemble saliva in its chemical composition, and there is an entire absence of the sulpho-cyanide of potash, which is so constantly found in true salivary secretion. Ranula situate in the submucous tissue is generally larger, and closely resembles sebaceous tumors, and contains a thick, putty-like substance, composed of epithelial scales, cholesterine, and oil globules. It is of rare occurrence, and the cyst walls are usually very thin and more or less adherent to the neighboring tissues. The removal of the entire sac and contents is the most satisfactory treatment where practicable. Ranula arising from an inflamed bursa should be treated on the principle of destroying the secreting surface, and thus curing the disease. Sanguineous or venous cysts are of rare occurrence in the mouth; when, however, they do occur, they are usually seen on the lips near to their free border. Sir James Paget regards them as "dilated portions of the blood vessels, shut off, as it were, from the main stream." They are usually congenital, and, as a rule, do not increase in size. When small and superficial they may be dissected out but when deep-seated a ligature or seton will often effect a cure. Cysts known as dentigerous cysts are usually confined to the alveolar portions of the maxillæ, and do not come within the scope of the present paper. Cysts formed in the tongue itself, and consisting of dilated mucous follicles, are difficult to diagnose, and may readily be mistaken for malignant disease. A puncture with a grooved needle will generally solve any doubt as to their nature. Their treatment consists in opening the sac and irritating the lining membrane by means of a probe or otherwise.—*Med. and Surg. Reporter.*

